# Challenges in Autologous Breast Reconstruction: A Review of Recommendations

**DOI:** 10.3390/jcm13040971

**Published:** 2024-02-08

**Authors:** Lauren M. Sinik, Meredith S. Collins

**Affiliations:** Department of Plastic Surgery, University of Kansas Medical Center, Kansas City, KS 66160, USA; lsinik@kumc.edu

**Keywords:** breast reconstruction, autologous breast reconstruction, DIEP flap

## Abstract

Breast reconstruction is an integral part of breast cancer treatment and offers significant psychosocial benefits for patients undergoing mastectomy as a part of their treatment plan. Autologous breast reconstruction (ABR) utilizes a patient’s own tissue to reconstruct the mastectomy defect, obviating the need for the implantation of a foreign object. As the field of plastic surgery progresses, ABR has become an excellent option for the recreation of a native breast mount. With that said, there are patient populations who present unique challenges when optimizing an aesthetic ABR result. We aim to discuss these challenging groups, including patients with both high and low body mass index (BMI), patients with a history of massive weight loss, patients who require post-mastectomy radiation therapy (PMRT), and patients electing for unilateral procedures where the surgeon attempts to match the reconstructed breast with the native breast. In our discussion, we review the literature recommendations for management as well as our experiences with our own patient cases. Ultimately, we believe this procedure can be performed in a wide variety of patient types and can be offered to those who may not be considered the “ideal” candidate.

## 1. Introduction

Breast reconstruction is an integral part of breast cancer treatment and offers significant psychosocial benefits for patients undergoing mastectomy as a part of their treatment plan [1]. There are two primary methods for post-mastectomy reconstruction; implant-based or autologous breast reconstruction. Implant-based reconstruction is the most common form of breast reconstruction in the United States, but it presents a significant risk of complications associated with the implanted device such as capsular contracture and rupture [2]. Autologous reconstruction, on the other hand, utilizes a patient’s own tissue to reconstruct the mastectomy defect, obviating the need for the implantation of a foreign object. Autologous reconstruction offers the benefits of a long-lasting result, tissue response to changes in patient weight, and donor site contouring [3]. Autologous reconstruction also offers the highest patient satisfaction of any breast reconstruction option [4,5,6].

Since the first autologous breast reconstruction by Verneuil in 1887, there has been profound progress within the field, making microsurgical autologous breast reconstruction more prevalent for the recreation of a native breast mound [4]. Abdominally based flaps evolved to become workhorse flaps for breast reconstruction due to their reliable blood supply and favorable donor site. Abdominally based free flaps for breast reconstruction include transverse rectus abdominis (TRAM) flaps, muscle-sparing TRAM (ms-TRAM) flaps, superficial inferior epigastric artery (SIEA), and deep inferior epigastric artery perforator (DIEP) flaps. At our institution, DIEP flaps are the most common procedure performed for autologous breast reconstruction as they allow for the transfer of abdominal skin and fat from the abdomen to the chest without sacrifice of rectus muscle or fascia [3].

While abdominally based free flaps are the primary focus of this paper, there are alternative donor site options for patients wishing to undergo autologous breast reconstruction who have a history of abdominal surgeries that may have disrupted the inferior epigastric system. If the integrity of the inferior epigastric system is in question, a CT angiogram (CTA) of the abdomen can be obtained to assist with the visualization of vessel continuity. Our senior author obtains abdominal CTAs on all patients pre-operatively to ensure the presence of DIEP vessels, as well as to map their locations prior to dissection. If the inferior epigastric system is not intact, donor site options include thigh-based flaps, including profunda artery perforator (PAP) flaps, lateral thigh flaps, diagonal upper gracilis (DUG) flaps, and transverse upper gracilis (TUG) flaps, gluteal-based flaps including superior gluteal artery perforator (SGAP) flaps and inferior gluteal artery perforator (IGAP) flaps, and finally lumbar artery perforator (LAP) flaps. While these alternative donor sites are not typically viewed as the first choice for autologous breast reconstruction, each has utility in specific subsets of patients. These flaps may also be used in conjunction with abdominally based free flaps in appropriate candidates.

The survival of a free flap in autologous breast reconstruction is paramount. Once flap viability is ensured, there are additional considerations in optimizing a patient’s result. Enhancing the aesthetic outcome of DIEP flaps has become the focus of our senior author’s practice. Our senior author has been a practicing microsurgeon with a focus on breast reconstruction for nine years. In 2023, she performed 77 DIEP flaps in 53 patients at a high-volume academic center with a total of five breast microsurgeons. With this substantial volume and focus on cosmetic outcomes, one becomes aware of patient groups that present unique challenges to successful, aesthetically appearing DIEP flap reconstruction. Our goal in this paper is to discuss these challenging groups and review the literature recommendations for management as well as our experiences with our own patient cases. The groups we have selected to review include patients with both high and low body mass index (BMI), patients with a history of massive weight loss, patients who require post-mastectomy radiation therapy, and patients electing for unilateral procedures where the surgeon attempts to match the reconstructed breast with the native breast.

## 2. High BMI

Increasing BMI is a well-studied risk factor for increasing complications in microsurgical autologous breast reconstruction. Ultimately, large studies have concluded that successful autologous breast reconstruction may be achieved in obese populations with no significant difference in flap survival, but with the risk of higher rates of donor and recipient site complications [7,8,9]. In a study of 3911 patients and a total of 4561 DIEP flaps, Heidekrueger et al. determined that overweight (BMI: 25–29.9 kg/m^2^) and obese (BMI ≥ 30 kg/m^2^) patients had significantly higher rates of postoperative infections at both the donor and recipient sites compared to the control group (BMI: 18.5–24.9 kg/m^2^) [8]. Obese patients in this study additionally had higher rates of medical complications postoperatively, including deep vein thrombosis, pulmonary embolism, and myocardial infarction [8]. Hassan et al. affirmed these findings in a study of 1250 patients with 1640 reconstructions, in which they found that patients with class II/III obesity (BMI ≥ 35 kg/m^2^) had higher rates of postoperative breast complications compared to patients with class I obesity (BMI 30–34.9 kg/m^2^) and non-obese patients [9]. Additionally, patients with any class of obesity had higher rates of donor site complications including infections, delayed wound healing, and seroma than did non-obese patients [9].

Given the findings of these studies in addition to others echoing the association of higher BMI with increased postoperative complications in autologous breast reconstruction, Barnes et al. set out to determine an optimal BMI cutoff based on surgical and outcome data from 365 patients and 545 reconstructions [7]. Ultimately, this group determined that a BMI cutoff of 32.7 and 30.0 kg/m^2^ would minimize the occurrence of any breast complication and any donor site complication, respectively [7].

Our senior author utilizes 35.0 kg/m^2^ as a BMI cutoff in her practice. A detailed preoperative discussion of surgical risks after DIEP flap reconstruction associated with increasing BMI is paramount. The aforementioned studies help facilitate these potentially challenging conversations. Referrals for medical or surgical weight loss prior to flap reconstruction are often utilized in our practice to help patients achieve their BMI goals. The importance of collaboration with our institution’s medical weight management and bariatric surgery groups cannot be overstated and is tremendously additive to our patients’ reconstructive journey.

DIEP flap breast reconstruction presents a challenge in patients with higher BMI not only due to an increased incidence of postoperative complications but also due to increased flap thickness, leading to difficulty with inset and the subsequent risk of poor aesthetic outcomes. Large volume DIEP flaps often require thinning or trimming prior to inset. It is important to consider the flap’s blood supply when thinning and shaping the flap to avoid compromising the flap vascularity while pursuing an improved aesthetic outcome. De la Parra-Marquez et al. describe a technique for thinning DIEP flaps in obese patients by resecting a progressively thicker wedge of the dermis and superficial fat from the flap in the area meant to recreate the upper pole [10]. This technique contrasts the previously described technique of deep fat removal, as the authors felt deep fat removal created a poorer aesthetic outcome with the development of a “step deformity” in the upper pole of the reconstructed breast [10].

Our senior author prefers the technique of De la Parra-Marquez at the time of the flap inset. If an overly full upper pole is still present at the time of the revision, a small area of focused liposuction can be used to further contour the transition from the chest wall to the reconstructed breast. Determining how much to remove with liposuction can be challenging, especially with the infiltration of tumescent solution. Care should be taken not to over-suction to avoid creating a contour irregularity. A preoperative discussion highlighting the potential need for additional revisions to achieve the desired outcome is essential. Re-elevation of the mastectomy skin with direct flap excision can also be used but does not allow for simultaneous fat grafting along the chest wall to improve the shape of the soft tissues around the edges of the flap. This technique is useful if fat necrosis along the superior edge of the flap is present and affects the upper pole contour and requires direct excision. In our senior author’s practice, patients are able to schedule DIEP revision procedures three months after their initial procedure. Most DIEP flap patients undergo a single revision surgery, with a smaller proportion of patients electing to undergo additional revisions.

## 3. Low BMI

After the discussion of challenges associated with DIEP flap autologous breast reconstruction in patients with higher BMIs, one might think that a patient with a lower BMI would allow for a simpler approach. This is not the case, however, as slender patients present a different set of challenges when it comes to autologous breast reconstruction as there may be a lack of tissue available for transfer. A patient in the BMI range of approximately 25–30 kg/m^2^ tends to be an excellent candidate for DIEP flap reconstruction due to the presence of redundant abdominal tissue for transfer, but not necessarily in excess. When considering bilateral breast reconstruction in slim patients, the standard DIEP flap may not be an ideal option if the patient has minimal abdominal bulk or redundancy. This may lead a surgeon to turn to alternative flap options such as thigh, gluteal, or lumbar flaps or to utilize a combination of flaps in a stacked fashion. Additional options include DIEP flap volume augmentation with delayed fat grafting or implant placement.

While slim patients may not traditionally be offered abdominally based bilateral autologous breast reconstruction due to a potential lack of tissue, Mani et al. hypothesized that enough tissue could be harvested in patients with BMI < 25 kg/m^2^ to perform a successful reconstruction with acceptable rates of complications [11]. Forty-two “slim” patients with a BMI of 20–24.9 kg/m^2^ were included in the study and were compared to “traditional” patients with a BMI of 25–29.9 kg/m^2^ and obese patients with BMI > 30 kg/m^2^ [11]. To assess the adequacy of tissue availability, mastectomy weight was compared to flap weight to yield a ratio. Bilateral DIEP reconstruction was successful in the slim patients, with similar tissue adequacy ratios in all groups [11].

Haddock et al. analyzed the outcomes following autologous breast reconstruction in patients with BMI < 23.5 kg/m^2^, including the traditional DIEP flap in addition to lumbar artery perforator (LAP) flaps, profunda artery perforator (PAP) flaps, and stacked flaps [12]. Stacked flap combinations for bilateral breast reconstruction included four flaps with DIEP and PAP flaps, four flaps with DIEP and LAP flaps, and four flaps with LAP and PAP flaps [12]. Ultimately, the group determined that autologous breast reconstruction may yield successful and durable results in low BMI patients with either the standard DIEP flap or alternate flap options [12]. While there were no flap losses in the group studied, the patients undergoing alternate flaps did experience more minor complications and donor site complications than the patients who underwent DIEP flap reconstruction [12].

The reviewed literature demonstrates that despite concern for lack of tissue availability in lower BMI patients, there are several options for autologous breast reconstruction that can create successful results in these patients. In addition to the initial flap procedure, revision procedures may assist in the volume augmentation of autologous breast reconstruction in patients with less tissue available for transfer. Laporta et al. presented their protocol for augmenting DIEP flap reconstructions with delayed large-volume fat grafting in patients with insufficient donor-site volumes, demonstrating high-scoring evaluations of aesthetic components of the final reconstruction by both surgeons and patients themselves [13]. Figus et al. also demonstrated the safety of utilizing a breast implant in conjunction with a DIEP flap reconstruction in either a primary or delayed fashion in order to optimize aesthetic results [14]. While it is an option, the use of an implant in an autologous breast reconstruction does negate some of the benefits of autologous breast reconstruction in that the patient assumes the risks associated with implant-based reconstruction in addition to those associated with autologous breast reconstruction.

In our practice, the senior author has performed flap augmentation with both fat grafting and implant placement. The preferred method in our practice has been multi-stage fat transfer which has yielded excellent results. Proper patient counseling that more than one round of fat grafting may be needed to achieve the desired result is key for setting proper patient expectations. Fat grafting volumes in our practice range from 100 to 300 cubic centimeters per reconstructed breast per session. Harvest is performed with power-assisted liposuction and 3 to 4 mm cannulas. Fat is grafted through incisions at the periphery of the breast with a 1.4 mm cannula.

## 4. Massive Weight Loss

Based on the prior discussion regarding patients with higher BMIs being at an increased risk of postoperative complications following abdominally based autologous breast reconstruction, some clinicians may encourage weight loss prior to offering these operations to their patients. Depending on the patient’s BMI, this weight loss strategy may include bariatric surgery or other, non-surgical weight loss methods. Massive weight loss is defined as a history of bariatric surgery or a history of intentional or unintentional weight loss greater than 50 pounds [15]. While massive weight loss may offer several benefits to a patient’s overall health, this weight loss may have the unintended consequence of having suboptimal effects on tissue. For example, the loss of large volumes of fat may attenuate subcutaneous support due to the atrophy of collagen and elastin in the extracellular matrix [16].

Our institution investigated outcomes of autologous breast reconstruction on patients with a history of massive weight loss, ultimately concluding that autologous breast reconstruction can be successfully undertaken in this population but may have a higher incidence of postoperative complications including delayed wound healing, surgical site infections, and partial flap losses as well as an increased need for donor and flap site revisions [17]. These conclusions were based on a cohort of 916 patients undergoing 1465 flaps, of which 39 patients had a history of massive weight loss [17]. Notably, this study also found that despite the increased risk of complications and increased need for surgical revisions, BREAST-Q scores were not statistically different between massive weight loss patients and patients without a history of weight loss with respect to satisfaction with breasts or satisfaction with the surgeon [17].

Similar results were seen in a study conducted by Bauder et al. comparing autologous breast reconstruction outcomes in 14 women who had undergone bariatric surgery prior to reconstruction to outcomes in 1012 patients without a history of bariatric surgery [18]. Bauder et al. determined that patients with a history of massive weight loss had a higher number of operative revisions, subsequent implant placements, and total operating room visits than patients without a history of massive weight loss [18]. An additional cohort study by Dayicioglu et al. compared 9 DIEP flaps performed on 6 patients with a history of massive weight loss to 141 DIEP flaps performed on 97 patients without a weight loss history [19]. After matching patients for age and BMI, this study noted no differences between the two groups in terms of flap failures, bulges, or hernias requiring operative revision, abdominal complications, hospitalization days, or operative time [19]. Based on the three studies discussed, it can be concluded that DIEP flaps may be successfully performed and yield a satisfactory result in patients with a history of massive weight loss, but these patients should likely be counseled on the potential for increased postoperative complications based on their history.

One additional finding in the literature regarding patients with a history of massive weight loss is the potential for increased utility of superficial inferior epigastric artery (SIEA) flaps in this population. Gusenoff et al. conducted a study examining 64 hemiabdomens in 32 patients undergoing abdominal contouring procedures following massive weight loss to characterize the diameter of the superficial inferior epigastric artery and vein in order to elucidate the feasibility of SIEA flaps in these patients [20]. Based on a threshold diameter of 1.5 mm, 51.6% of the arteries and 90.6% of the veins were designated as being usable for a SIEA flap autologous breast reconstruction, with the presence of usable vessels being associated with both maximum and current BMI [20]. The correlation between maximum BMI and vessel size could suggest that patients with a history of massive weight loss may have a higher incidence of vessels appropriately sized for an SIEA flap reconstruction than those without this history. These findings were reinforced in the previously discussed study by Bauder et al., in which SIEA faps comprised 19.2% of the flaps performed in the patients with a history of bariatric surgery compared to only 6.5% of the flaps performed in the control group without a history of massive weight loss [18]. This is notable as SIEA flaps confer some advantages over DIEP flaps as they do not require incision or excision of the rectus abdominis muscle and fascia and may, therefore, contribute to lower rates of donor-site morbidity [20].

Our senior author routinely performs abdominally based free flap breast reconstruction on massive weight loss patients. As stated previously, we utilize a body mass index goal of 35 mg/kg^2^ for DIEP flap surgery and thus our patients who initially present with higher BMIs may fall into this category after medical or surgical weight loss. Since the abdominal skin excess may be more than is needed to reconstruct the breast, we will take the width of tissue required for the flap volume and excise more abdominal skin during closure if a significant abundance still remains. We have found that patient satisfaction is generally very high in this population as DIEP flap surgery is transformative for both the breasts and abdomen.

## 5. Radiation

Post-mastectomy radiation therapy is an important facet of breast cancer treatment and can impact reconstructive efforts in multiple ways. One challenge in performing autologous breast reconstruction on patients requiring post-mastectomy radiation therapy is the determination of the optimal timing for the reconstruction. Studies have been conducted to investigate the outcomes of autologous breast reconstruction when performed prior to post-mastectomy radiation therapy versus after the completion of post-mastectomy radiation therapy. O’Connell et al. investigated the aesthetic outcomes and patient satisfaction in patients undergoing both post-mastectomy radiation therapy and DIEP flap reconstructions and utilized patients undergoing DIEP flaps without post-mastectomy radiation therapy as a control group [21]. The study group included patients undergoing immediate DIEP flap reconstruction with post-mastectomy radiation therapy, patients with an initial mastectomy and short-term implant placement followed by post-mastectomy radiation therapy with delayed DIEP reconstruction, and patients with a simple mastectomy, post-mastectomy radiation therapy, and delayed DIEP reconstruction [21]. In this study group, patients who avoided post-mastectomy radiation therapy altogether were the most satisfied with their results overall; however, in patients who required post-mastectomy radiation therapy, those undergoing delayed reconstruction after simple mastectomy were the most satisfied [21]. There was no difference in satisfaction between those who had post-mastectomy radiation therapy following immediate DIEP versus those who had post-mastectomy radiation therapy following implant placement and delayed DIEP reconstruction, suggesting that both are viable treatment pathways [21].

In those requiring post-mastectomy radiation therapy after DIEP flap reconstruction, it may be difficult to predict the effects of radiation on the final aesthetic outcome. Craig et al. investigated this by analyzing DIEP flap volume, projection, and position in 11 patients following immediate bilateral DIEP flap reconstructions with unilateral post-mastectomy radiation therapy [22]. The group noted that there were no changes in flap volume or projection of the DIEP flap following post-mastectomy radiation therapy, but the position of the irradiated DIEP flap was higher on the chest wall compared to the non-irradiated side [22]. Patient satisfaction with their reconstruction also decreased following post-mastectomy radiation therapy, though only 20% said they would have preferred to delay their reconstruction for an improved aesthetic result [22].

While the effect of post-mastectomy radiation therapy on a DIEP flap reconstruction may change the aesthetic outcome and decrease satisfaction, the effects of radiation on the tissue quality can also present challenges for the reconstruction if autologous breast reconstruction is performed after the completion of post-mastectomy radiation therapy. A large study on 3926 patients with 4577 DIEP flaps by Prantl et al. examined the outcomes in patients who had post-mastectomy radiation therapy prior to their DIEP flap reconstruction compared to patients who did not require radiation and found that patients with a history of radiation had increased risk of wound-healing complications at their recipient sites [23]. While this complication increased, the groups did not have significant differences in the rates of total flap loss, partial flap loss, or revision surgeries, suggesting that post-radiation-free flap reconstruction is safe and feasible [23]. Godden et al. further examined the impact of pre-operative radiation on DIEP flap reconstruction by evaluating the aesthetic outcomes of these flaps compared to DIEP flaps which were radiated [24]. In this study, the aesthetic outcome was rated as ‘good’ or ‘excellent’ in 93% of patients who underwent pre-operative radiation and delayed DIEP flap reconstruction, with patient satisfaction being significantly better compared to patients receiving post-mastectomy radiation therapy following immediate DIEP reconstruction at 12 months [24].

At our institution, the typical protocol for patients who require post-mastectomy radiation therapy is the placement of a tissue expander or implant at the time of mastectomy and delayed autologous reconstruction at least six months following radiation. If the flap is of sufficient size, the senior author prefers to excise a portion of the radiated skin and resurface the lower pole of the breast for improved ptosis and symmetry with the non-radiated side. We leave a 1 cm cuff of skin along the inframammary fold to prevent a stuck-on appearance. Maintaining symmetry in bilateral DIEP flap reconstruction in the setting of unilateral post-mastectomy radiation is a major challenge as the non-radiated side will descend more quickly than a naturally aging breast would. Proper counseling on the lack of stretch over time of the radiated skin and decreasing symmetry as the patient ages is important. A revision procedure to lift the non-radiated breast may be offered years after the initial reconstruction once the degree of asymmetry becomes intolerable.

## 6. Unilateral

The final group that we wish to discuss who present unique challenges with respect to autologous breast reconstruction are those who undergo unilateral autologous breast reconstruction. These reconstructions may be challenging due to the difficulty in creating a symmetrical result between a native breast and a reconstructed breast. There are a multitude of native breast phenotypes and additional variability in donor site characteristics which make approaching a unilateral reconstruction unique for each patient. Techniques that may help achieve a symmetrical result include flap inset techniques, contralateral symmetry procedures such as breast reduction or mastopexy, or revision procedures to reconstructed breasts including fat grafting or other forms of tailoring.

Razzano et al. published an algorithm with an approach for optimizing DIEP flap inset in immediate unilateral reconstruction, accounting for both donor site variables including perforator selection, venous supercharge, use of a bipedicled flap, scars, and tissue thickness as well as breast variables including ptosis, weight, and use of contralateral symmetry procedures [25]. With these variables, the algorithm assists providers in determining the direction and degree of flap rotation for an ideally aesthetic inset [25].

There are additional techniques reported specifically in the setting of slim patients undergoing unilateral autologous breast reconstruction when volume augmentation is required. In these cases, it may be useful to design a flap that crosses over the patient’s midline in order to recruit additional tissue. An additional consideration is the use of a bipedicled flap or an abdominally based flap that is based on two pedicles, either both from the deep inferior epigastric, both from the superficial inferior epigastric, or a combination of the two. Pompei et al. presented a technique for using a bipedicled “calzone” flap for autologous breast reconstruction of medium/large size breasts in patients with minimal abdominal excess or previous abdominal scars which may limit the harvest of an entire pannus on a single perforator [26]. The authors advocate for folding over the flap following its harvest, creating a configuration where the two pedicles are close to one another and aligned towards the chest recipient vessels, ultimately connecting the pedicles to the anterograde and retrograde limbs of the internal mammary vessels [26]. This technique was utilized in 28 patients with no flap failures [26].

In our practice, the senior author routinely crosses the midline on the abdomen if the vasculature allows or utilizes a bipedicle flap for additional volume and skin to improve symmetry with the contralateral native breast. Prior to crossing the midline, perfusion of the flap is tested on the selected perforator(s) to evaluate the amount of the contralateral hemiabdomen that can be utilized. This is particularly helpful in resurfacing the lower pole of a radiated breast as having additional skin from the contralateral hemiabdomen can create a more rounded and naturally ptotic breast. To optimize aesthetic outcomes, the senior author also prefers to delay all symmetry procedures, such as breast reduction, mastopexy, and fat grafting, until a second stage revision is carried out to allow the flap to settle. Revision is performed at least three months after the initial flap surgery. Expectation setting for revision procedures is also key, as more than one may be necessary to achieve the optimal aesthetic outcome.

## 7. Conclusions

Autologous breast reconstruction, and in particular DIEP flap breast reconstruction, is an excellent option for breast cancer reconstruction in a wide variety of patients and can offer superior aesthetic results when compared to alternate forms of breast reconstruction. As DIEP flap reconstruction has become more commonplace, it has been referred to as the “gold standard” for the restoration of a breast mound and often carries high expectations given this reputation. There are certain patient populations that create unique challenges for the creation of a highly cosmetic result in DIEP flap reconstruction, and we wished to review these groups and the recommendations for the optimization of the results in these populations along with a narrative highlighting our experience. While the evidence is ultimately limited, we believe this procedure can be performed in a wide variety of patient types and can be offered to those who may not be considered the “ideal” candidate. As with any surgical procedure, a detailed preoperative discussion is of key importance to set proper expectations for the reconstruction timeline, anticipated outcomes, and the potential need for future revisions to maintain a symmetric result.

## References

[1] Kaya B., Serel S. (2013). Breast reconstruction. Exp. Oncol..

[2] Stefura T., Rusinek J., Wątor J., Zagórski A., Zając M., Libondi G., Wysocki W.M., Koziej M. (2023). Implant vs. autologous tissue-based breast reconstruction: A systematic review and meta-analysis of the studies comparing surgical approaches in 55,455 patients. J. Plast. Reconstr. Aesthetic Surg. JPRAS.

[3] Costanzo D., Klinger M., Lisa A., Maione L., Battistini A., Vinci V. (2020). The evolution of autologous breast reconstruction. Breast J..

[4] Macadam S.A., Bovill E.S., Buchel E.W., Lennox P.A. (2017). Evidence-Based Medicine: Autologous Breast Reconstruction. Plast. Reconstr. Surg..

[5] Toyserkani N.M., Jørgensen M.G., Tabatabaeifar S., Damsgaard T., Sørensen J.A. (2020). Autologous versus implant-based breast reconstruction: A systematic review and meta-analysis of Breast-Q patient-reported outcomes. J. Plast. Reconstr. Aesthetic Surg. JPRAS.

[6] Santosa K.B., Qi J., Kim H.M., Hamill J.B., Wilkins E.G., Pusic A.L. (2018). Long-term Patient-Reported Outcomes in Postmastectomy Breast Reconstruction. JAMA Surg..

[7] Barnes L.L., Lem M., Patterson A., Segal R., Holland M.C., Lentz R., Sbitany H., Piper M. Relationship Between BMI and Outcomes in Microvascular Abdomen-Based Autologous Breast Reconstruction. Plast. Reconstr. Surg..

[8] Heidekrueger P., Fritschen U., Moellhoff N., Germann G., Giunta R., Zeman F., Prantl L. (2021). Impact of body mass index on free DIEP flap breast reconstruction: A multicenter cohort study. J. Plast. Reconstr. Aesthetic Surg. JPRAS.

[9] Hassan A.M., Paidisetty P., Ray N., Govande J.V., Largo R.D., Chu C.K., Mericli A.F., Schaverien M.V., Clemens M.W., Hanasono M.M. (2023). Ensuring Safety While Achieving Beauty: An Evidence-Based Approach to Optimizing Mastectomy and Autologous Breast Reconstruction Outcomes in Patients with Obesity. J Am Coll Surg..

[10] De la Parra-Marquez M., Fernandez-Riera R., Romay-Chambers E., Escamilla Linaje T. (2021). Superficial Thinning of the DIEP Flap: A Safe Option to Achieve an Aesthetic Reconstructed Breast in the Obese Patient. Plast. Reconstr. Surg..

[11] Mani M., Saour S., Ramsey K., Power K., Harris P., James S. (2018). Bilateral breast reconstruction with deep inferior epigastric perforator flaps in slim patients. Microsurgery.

[12] Haddock N.T., Mejia Martinez V., Teotia S.S. (2023). Surgical Outcomes of Autologous Breast Reconstruction in Low BMI Patients; Beyond the Gold Standard DIEP Flap. Plast. Reconstr. Surg..

[13] Laporta R., Longo B., Sorotos M., Pagnoni M., Santanelli di Pompeo F. (2015). Breast Reconstruction with Delayed Fat-Graft-Augmented DIEP Flap in Patients with Insufficient Donor-Site Volume. Aesthetic Plast. Surg..

[14] Figus A., Canu V., Iwuagwu F.C., Ramakrishnan V. (2009). DIEP flap with implant: A further option in optimising breast reconstruction. J. Plast. Reconstr. Aesthetic Surg. JPRAS.

[15] Wakefield W., Rubin J.P., Gusenoff J.A. (2014). The life after weight loss program: A paradigm for plastic surgery care after massive weight loss. Plast. Surg. Nurs. Off. J. Am. Soc. Plast. Reconstr. Surg. Nurses.

[16] Singh D., Forte A.J.V., Zahiri H.R., Janes L.E., Sabino J., Matthews J.A., Bell R.L., Thomson J.G. (2012). Prognostication for body contouring surgery after bariatric surgery. Eplasty.

[17] Sinik L.M., Elver A.A., Egan K.G., Johnson B.M., Cullom M.E., Limpiado M., Nazir N., Lai E.C., Butterworth J.A.M. (2023). Autologous Breast Reconstruction after Massive Weight Loss: Understanding Risks in a Growing Population. Plast. Reconstr. Surg..

[18] Bauder A.R., Samra F., Kanchwala S.K., Serletti J.M., Kovach S.J., Wu L.C. (2018). Autologous breast reconstruction in the postbariatric patient population. Microsurgery.

[19] Dayicioglu D., Tugertimur B.B., Munzenmaier K.B., Khan M.B., Smith P., Murr M., Kumar A., Khakpour N. (2016). Outcomes of Breast Reconstruction After Mastectomy Using Deep Inferior Epigastric Perforator Flap After Massive Weight Loss. Ann. Plast. Surg..

[20] Gusenoff J.A., Coon D., De La Cruz C., Rubin J.P. (2008). Superficial inferior epigastric vessels in the massive weight loss population: Implications for breast reconstruction. Plast. Reconstr. Surg..

[21] O’connell R.L., Di Micco R., Khabra K., Kirby A.M., Harris P.A., James S.E., Power K., Ramsey K.W.D., Rusby J.E. (2018). Comparison of Immediate versus Delayed DIEP Flap Reconstruction in Women Who Require Postmastectomy Radiotherapy. Plast. Reconstr. Surg..

[22] Craig E.S., Lentz R., Srinivasa D., Chuang C., Walker M.E., Higgins S.A., Salomon J., Fusi S. (2018). Three-dimensional Analysis of How Radiation Affects Deep Inferior Epigastric Perforator (DIEP) Flap Volume, Projection, and Position in Breast Cancer Reconstruction. Ann. Plast. Surg..

[23] Prantl L., Moellhoff N., von Fritschen U., Giunta R., Germann G., Kehrer A., Thiha A., Ehrl D., Zeman F., Broer P.N. (2021). Effect of Radiation Therapy on Microsurgical Deep Inferior Epigastric Perforator Flap Breast Reconstructions: A Matched Cohort Analysis of 4577 Cases. Ann. Plast. Surg..

[24] Godden A.R., Micha A., O’Connell R.L., Mohammed K., Kirby A.M., Thiruchelvam P.T., Leff D.R., MacNeill F.A., Rusby J.E., Cleator S. (2023). Pre-operative Radiotherapy And Deep Inferior Epigastric Artery Perforator (DIEP) flAp study (PRADA): Aesthetic outcome and patient satisfaction at one year. J. Plast. Reconstr. Aesthetic Surg. JPRAS.

[25] Razzano S., Marongiu F., Wade R., Figus A. (2019). Optimizing DIEP Flap Insetting for Immediate Unilateral Breast Reconstruction: A Prospective Cohort Study of Patient-Reported Aesthetic Outcomes. Plast. Reconstr. Surg..

[26] Pompei B., Farhadi J. (2020). Diep Flap Volume Augmentation: Literature Review and “Calzone” Flap Shaping Technique. J. Plast. Reconstr. Aesthetic Surg. JPRAS.

